# Combination Treatment of Omega-3 Fatty Acids and Vitamin C Exhibited Promising Therapeutic Effect against Oxidative Impairment of the Liver in Methotrexate-Intoxicated Mice

**DOI:** 10.1155/2022/4122166

**Published:** 2022-04-20

**Authors:** Mohammed Alorabi, Doha Saad Mohammed, Gomaa Mostafa-Hedeab, Suzy A. El-Sherbeni, Walaa A. Negm, Ali Ismail A. Mohammed, Hayder M. Al-kuraishy, Nani Nasreldin, Saqer S. Alotaibi, Bashir Lawal, Gaber El-Saber Batiha, Carlos Adam Conte-Junior

**Affiliations:** ^1^Department of Biotechnology, College of Sciences, Taif University, P.O.Box 11099, Taif 21944, Saudi Arabia; ^2^Department of Clinical Pharmacology, College of Medicine, University of Al-Mustansiriyah, Iraq; ^3^Pharmacology Department & Health Research Unit, Medical College, Jouf University, Jouf, Saudi Arabia; ^4^Pharmacology Department–Faculty of Medicine, Beni-Suef University, Egypt; ^5^Department of Pharmacognosy, Faculty of Pharmacy, Tanta University, Tanta 31111, Egypt; ^6^Department of Pathology and Clinical Pathology, Faculty of Veterinary Medicine, New Valley University, El-Kharga, P.O. Box 72511, Egypt; ^7^PhD Program for Cancer Molecular Biology and Drug Discovery, College of Medical Science and Technology, Taipei Medical University and Academia Sinica, Taipei 11031, Taiwan; ^8^Graduate Institute for Cancer Biology & Drug Discovery, College of Medical Science and Technology, Taipei Medical University, Taipei 11031, Taiwan; ^9^Department of Pharmacology and Therapeutics, Faculty of Veterinary Medicine, Damanhour University, Damanhour 22511, Egypt; ^10^Center for Food Analysis (NAL), Technological Development Support Laboratory (LADETEC), Federal University of Rio de Janeiro (UFRJ), Cidade Universitária, Rio de Janeiro 21941-598, Brazil

## Abstract

Drug-induced liver injury (DILI) is the main cause of liver damage mediated by the excretion of toxic active drug metabolites. Omega-3 fatty acids and vitamin C have potent antioxidant, anti-inflammatory, and antiapoptotic effects that could offer protection against oxidative stress and liver damage. This study evaluated the hepatoprotective effect of omega-3 and vitamin C alone as well as in a combined form in methotrexate- (MTX-) induced acute liver injury in mice. Male ICR mice of seven groups (7 mice per group) were used. Groups 1 (control group) and 2 (MTX) received 0.9% saline/day (po) for 9 days. Groups 3 and 4 received 100 and 200 mg/kg bw/day omega-3 (po), respectively, for 9 days. Groups 5 and 6 received 100 and 200 mg/kg bw/day vitamin C (po), respectively, for 9 days, while group 7 received omega-3 (100 mg/kg bw/day) and vitamin C (100 mg/kg bw/day) (po) for 9 days. All animals in groups 2 to 7 received 20 mg/kg/day MTX (I.P.) once on the 10^th^ day. Our results revealed that MTX significantly induced the elevation of transaminases, alkaline phosphates (ALP), lactate dehydrogenase (LDH), and malonaldehyde (MDA) while depleting the levels of superoxide dismutase (SOD) and glutathione (GSH) when compared to the control group. Treatment with omega-3 fatty acids or vitamin C significantly attenuated the antioxidants and biochemical alterations in a dose-independent manner. Our molecular docking study of ligand-receptor interaction revealed that both ascorbic acid and omega-3 docked well to the binding cavity of LDH with high binding affinities of –5.20 and –4.50 kcal/mol, respectively. The histopathological features were also improved by treatment with omega-3 and vitamin C. The combined form of omega-3 and vitamin C showed a remarkable improvement in the liver enzymes, oxidative stress biomarkers, and the histopathological architecture of the mice. Conclusively, the combination of omega-3 and vitamin C demonstrated a synergistic therapeutic effect against MTX-intoxicated mice, hence representing a potential novel strategy for the management of drug-induced liver disorders.

## 1. Introduction

Drug-induced liver injury (DILI) is a global burden with an incidence rate of about 14~19 cases per 100,000 humans, with 30% of conditions accompanied by jaundice [1]. In Western countries, DILI accounts for 3 to 5% of patients admitted to hospital due to acute liver injury and jaundice [2]. The liver is one of the most important organs that play a central role in the metabolism of drugs, xenobiotic, and nutrients [3] and hence is highly susceptible to drug-induced damage [4]. Liver damage could be acute or chronic. The acute liver injury could be attenuated via rapid elimination of toxic agents and maintain normal liver function [5]; however, an untreated acute liver injury could progress to liver cirrhosis, fibrosis, encephalopathy, and liver cancer [6]. Drug-induced liver damage is associated with the generation of reactive oxygen species (ROS) and reactive nitrogen species (RNS) [7] coupled with the depletion of endogenous antioxidants including the superoxide dismutase (SOD), reduced glutathione (GSH), and catalase (CAT) leading to the condition known as oxidative stress and lipid peroxidation [8].

Methotrexate (4-amino-10-methyl folic acid, MTX) is a competitive irreversible inhibitor of dihydrofolate reductase (DHFR) [9] that is effectively used in the treatment of cancer, rheumatoid arthritis, and other inflammatory diseases [10–12]. However, the efficacy of this drug is associated with adverse effects, especially on the liver [13–16]. The underlying mechanism of MTX-induced hepatotoxicity has not been fully understood [17]; however, distinct mechanisms of action have been proposed including deregulation of cellular antioxidant defense which cause increased generation of free radicals and oxidative stress-induced damages to the hepatocyte [18]. Free radical scavengers including CAT, SOD, GSH, and GST continuously eliminate the ROS and maintain the steady mitochondrial function [19, 20]. However, MTX causes liver mitochondrial damage via depletion of these enzymatic and nonenzymatic antioxidants system, allowing the accumulation of ROS, and induction of oxidative stress [21]. MTX decreases the intracellular levels of NADPH by inhibiting the cytosolic NADP-dependent dehydrogenase and NADP-malic enzyme [13]. NADPH is essential for the activities of glutathione reductase (GR), an enzyme that sustains the levels of GSH. Thus, the decrease in the levels of GSH due to MTX leads to a suppression of the antioxidant defense system that protects the hepatocyte against free radicals [13]. In addition, MTX is stored in polyglutamated form in the hepatocytes, and its accumulation leads to decrease folate levels; hence, increased levels of polyglutamates are considered an important mechanism in the hepatotoxic effect of MTX [13].

In addition to the induction of oxidative and liver damage, methotrexate has also been implicated in renal oxidative stress and kidney impairment [21, 22]. High-dose MTX produces a wide clinical range of kidney damages ranging from subclinical tubulopathy to acute renal failure [23]. It has been reported that MTX induced tissue haemorrhages, tubular cell necrosis, inflammatory cell infiltrations, glomerulosclerosis [22], and significant alterations to the serum biochemical indices of the liver [24] as well as kidney damage [25].

Omega-3 fatty acids are polyunsaturated fatty acids (PUFAs) with health-promoting activities such as antioxidant [26], anti-inflammatory [27], and immunomodulation activities [28]. Vitamin C (L-ascorbic acid) is a known antioxidant agent with several biological activities against different diseases [29]. It exhibited antioxidants [30] and anti-inflammatory [31] activities with potential inhibitory roles against proinflammatory cytokines [32], chemokines, TNF-*α*, NF-*κ*B, IL-1, and IL-6 [33]. It also exhibited neuroprotective roles against toxicants that induced neuronal impairment [34]. It has also been reported to exhibit anticancer and immunomodulatory activities [35, 36]. The current study was designed to assess the potential hepatoprotective effect of different doses of omega-3 fatty acids, vitamin C, and the combined treatment (omega-3+vitamin C) against MTX-induced acute liver injury in mice. The histopathological changes and serum levels of ALT, ALP, AST, and liver LDH as well as oxidative stress markers including MDA, SOD, and GSH were analyzed.

## 2. Materials and Methods

### 2.1. Drugs, Assay Kits, and Reagents

The drugs and chemicals used including methotrexate, omega-3, vitamin C, malonaldehyde (MDA), mouse lactate dehydrogenase (LDH), mouse superoxide dismutase (SOD), and mouse glutathione (GSH) kits were purchased from MyBioSource, USA.

### 2.2. Drug Preparations

The MTX ampoule (50 mg/5 ml) injection used to induce hepatotoxicity in experimental mice was administered at an equivalent dose of 2 ml/kg (20 mg/kg/day, intraperitoneal (I.P.) [35]. Vitamin C powder sachet 1000 mg was dissolved in distilled water and administered orally to the experimental mice by using oral gavage at doses of 100 and 200 mg/kg [34]. Omega-3 soft gelatin capsule 1000 mg which contains eicosapentaenoic acid (EPA, 360 mg) and docosahexaenoic acid (DHA, 240 mg) was diluted with 5 ml dimethyl sulfoxide solution 5% (DMSO 5%). The solution was administered orally by using oral gavage at doses of (100 mg/kg and 200 mg/kg)/day [33].

### 2.3. Experimental Animals

A total of forty-nine (49) male ICR mice (average weight of 25.92 ± 1.35 g, 7 weeks) were obtained from the Animals Center of Al-Mustansiriyah University, Iraq. The mice were kept under a 12 hr light/dark cycle and under standard laboratory conditions (temperature of 25 ± 2°C and relative humidity of 60–65%). They were kept under standard conditions for acclimatization for 14 days before being studied. Animal experimentation was conducted in compliance with the Affidavit of Approval of animal use protocol (approval no. ICCMGR-2020-016) issued by the Ethics Committee on Animals Use of the Department of pharmacology, Collage of Medicine, Al-Mustansiriyah University, and the Iraqi Center of Cancer Research and Medical Genetics (ICCMGR), Al-Mustansiriyah University, Baghdad, Iraq. This is in line with the internationally accepted principles for laboratory animal use and care as contained in the Canadian Council on Animal Care (CCAC) guidelines on animal use protocol review.

### 2.4. Experimental Design and Drug Administrations

The experimental mice were randomly divided into 7 groups (*n* = 7 mice per group) as described in Table 1. All oral treatments (o.p) were administered with the aid of an oesophagal cannula for 9 days. MTX was administered to animals in groups 2-7 only on the 10th day via intraperitoneal (I.P.) injection at a single dose of 20 mg/kg bw/day [37]. The detailed experimental grouping and timeline are shown in Table 1.

### 2.5. Sample Collections and Processing

The animals were allowed for 48 hours after MTX injection and were sacrificed under diethyl ether anaesthesia. The procedures described by Shittu et al. [38] were adopted for the sample collections and preparations. The blood samples were collected via heart puncture into a clean, dry centrifuge tube. The blood samples were allowed to clot for 30 min and thereafter centrifuged at 4000 rpm for 5 minutes at 25°C [39]. The resulting sera were aspirated and kept frozen at -20°C till used for biochemical analysis [40]. Furthermore, the mice were quickly dissected, and the liver was isolated and weighed. A known weight (0.3 g) of the liver was homogenized in an ice-cold protein extraction solution (1 : 5 *w*/*v*). The homogenates were further centrifuged for 10 minutes at 5000 rpm after which the resulting supernatants were used immediately for the oxidative tissue analysis [41, 42]. The remaining liver tissue was fixed with formalin 10% for histopathological analysis.

### 2.6. Analysis of Liver Oxidative Stress Markers

The analyses of liver malonaldehyde (MDA), superoxide dismutase (SOD), glutathione (GSH), and lactate dehydrogenase (LDH) were conducted using ELISA kits according to the manufactural instruction. The principle of MDA analysis was based on double antibody sandwich technique. Analysis of superoxide dismutase (SOD) activities was based on the principle of the quantitative sandwich enzyme immunoassay technique, while the determination of glutathione (GSH) level was based on the principle test of the competitive double antibody sandwich technique. The prepared reaction mixture was monitored at the wavelength 450 nm using the ELISA microplate HumaReader (ELISA microplate HumaReader).

### 2.7. Analysis of Serum Enzymes of Hepatocellular Markers

All serum biochemical analyses were conducted using Randox Diagnostics kit and by using automated biochemical analyzer. Alanine transaminase (ALT) was analyzed based on the principle of catalytic action of ALT on alanine and *α*–oxoglutarate to form pyruvate and glutamate [43]. Aspartate transaminase (AST) was measured by monitoring the concentration of oxaloacetate hydrazone formed with 2,4-dinitrophenyl hydrazine [44].

### 2.8. Histopathological Analysis

The liver tissue was prepared for microscopic evaluation of the liver histology by the conventional procedure known as the paraffin-embedded process [45]. The liver sections (0.4~0.5 *μ* thickness) were further stained with hematoxylin and eosin (H&E) to visualize the overall morphology. The images were captured at ×100 using a light microscope equipped with digital camera software. The photomicrographs of the tests were compared with the control and the MTX-intoxicated group for any histoarchitectural changes. The liver tissue was ranked for lesion severity based on a semi-quantitative scoring of 0~3 depending on the degree and extent of the alteration [37].

### 2.9. Molecular Docking Studies

The three-dimensional (3D) structure of the lactate dehydrogenase (PDB: 1I10) was downloaded from the protein data bank (PDB). The PDB files of the crystal structures of the targets were transformed to pdbqt format using AutoDock Vina [46]. Ligand and receptor preparations for docking were conducted as described previously [47, 48], while molecular docking was performed using AutoDock Vina [46] following the protocols described in the previous studies [49–51]. The docked complexes were visualized with the Discovery Studio visualizer version [52].

### 2.10. Statistical Analysis

Data were analyzed using the statistical package for the social sciences software (SPSS Version 16) and presented as mean ± standard deviation (SD) of the replicate samples (*n* = 6). A one-way analysis of variance (ANOVA) was used for comparing the differences among treatment groups. An unpaired *t*-test was used to evaluate the statistical significance between individual groups. Statistically significant differences are represented as ∗*p* < 0.05, ∗∗*p* < 0.001, and ∗∗∗*p* < 0.001.

## 3. Results

### 3.1. Omega-3 Fatty Acids and Vitamin C Synergistically Improve the Antioxidant Status and Biochemical Alterations in Methotrexate-Induced Hepatotoxic Mice

**Figure 1:**
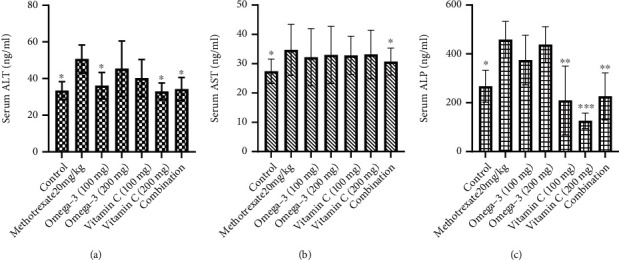
Effects of omega-3 fatty acids and vitamin C on serum levels of (a) ALT, (b) AST, and (c) ALP in methotrexate-induced hepatotoxic mice. ALT: alanine aminotransferase; AST: aspartate aminotransferase; ALP: alkaline phosphatase. ∗*p* < 0.05, ∗∗*p* < 0.01, and ∗∗*p* < 0.001.

**Figure 2:**
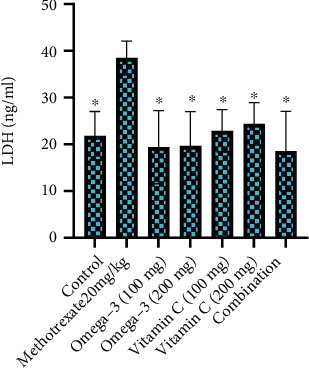
Effects of omega-3 fatty acids and vitamin C on hepatic lactate dehydrogenase (LDH) activities in methotrexate-induced hepatotoxic mice. ∗*p* < 0.05.

**Figure 3:**
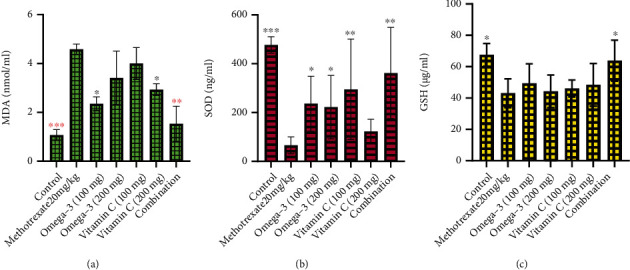
Effects of omega-3 fatty acids and vitamin C on hepatic malonaldehyde (MDA) and antioxidant enzymes in methotrexate-induced liver impairment. MDA: malonaldehyde; SOD: superoxide dismutase; GSH: glutathione. ∗*p* < 0.05, ∗∗*p* < 0.01, and ∗∗*p* < 0.001.

The results of the present study revealed that the intraperitoneal injection of MTX (20 mg/kg) significantly induced the elevation of serum transaminases and ALP (Figure 1(a)), hepatic LDH (Figure 2), and MDA (Figure 3(a)) while depleting the activities of antioxidants enzymes including SOD and GSH (Figure 3(c)) when compared to the control group. Treatment of the MTX-intoxicated mice with omega-3 fatty acids or vitamin C at 100 and 200 mg/kg BW significantly attenuated the antioxidants and biochemical impairments in a dose-independent manner. However, mice treated with a combination of omega-3 fatty acids and vitamin C at lower doses exhibited higher protective effects when compared with the individual therapy. Collectively, our results demonstrated that the omega-3 fatty acids and vitamin C synergistically improve the antioxidant status and biochemical alterations in MTX-induced hepatotoxic mice.

### 3.2. Omega-3 Fatty Acids and Vitamin C Synergistically Ameliorated the Liver Histoarchitectural Abnormalities in Methotrexate-Induced Hepatotoxic Mice

**Figure 4:**
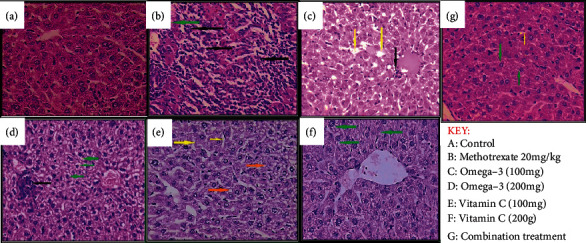
Liver histopathological section of methotrexate-induced liver-impaired mice treated with omega-3 and vitamin C. Stained with H and E ×40.

The liver histopathological changes mediated by MTX and the therapeutic effects of omega-3 fatty acids and/or vitamin C in reversing the compromised liver histological architecture were evaluated [15]. Our results revealed that in comparison with the control mice which shows the normal hepatic architecture and normal lobular rearrangement (Figure 4(a)), the liver section of the MTX-intoxicated mice showed severe hepatic tissue necrosis (Figure 4(b)). Treatment with omega-3 (100/kg mg) (Figure 4(c)) induced the amelioration of hepatoarchitectural alterations (from severe to mild changes) and reduced severity of hepatic congestion and inflammatory cell infiltration that were observed in the MTX-treated group, but when the dose of omega-3 pretreated increases to 200 mg/kg, the liver section (Figure 4(d)) showed moderate histopathological finding compared to MTX-treated group. Similarly, mice treated with vitamin C (100 and 200 mg/kg) showed mild histopathological changes compared with the MTX-treated group (Figures 4(e) and 4(f)). The combination of pretreatment (vitamin C+omega-3) 100 mg/kg (Figure 4(g)) provided very mild sinusoidal dilatation and very mild hepatic degenerative changes compared with the MTX group (Figure 4 and Table 2). Altogether, our results revealed that the omega-3 fatty acids and vitamin C synergistically preserved the liver histoarchitectural integrity of MTX-intoxicated mice.

### 3.3. Molecular Docking Revealed Higher Affinity and Target Ligandability of Ascorbic Acid for Lactate Dehydrogenase

**Figure 5:**
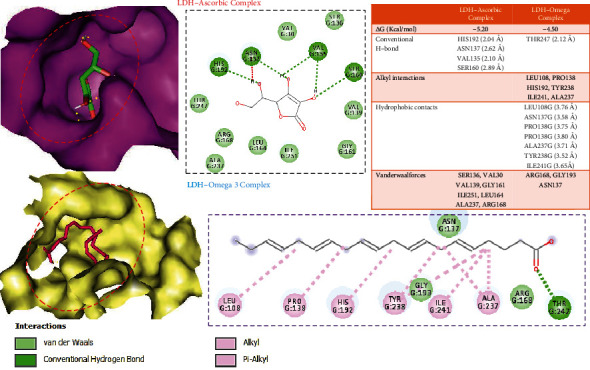
Docking profile of ascorbic acid/omega-3 with the binding cavity of lactate dehydrogenase.

Using in silico ligand-receptor interaction model, we found that both ascorbic acid and omega-3 docked well to the binding cavity of LDH with the high binding affinities of –5.20 and –4.50 kcal/mol, respectively. Ascorbic acid is bounded by four hydrogen bonding to the HIS192 (2.04 Ӑ), ASN137 (2.62 Ӑ), VAL135 (2.10 Ӑ), and SER160 (2.89 Ӑ) residues of LDH binding cavity, while the hydrogen bonding with THR247 (2.12 Ӑ) of the LDH binding site exists between LDH-omega complex. Interestingly, all the conventional hydrogen interactions between LDH and ascorbic acid/omega-3 occur at very close proximity (<3.0 Ӑ). Omega-3 is also bound to LDH by several alkyl and hydrophobic contacts. In addition, LDH-ascorbic acid/omega-3 complexes were strengthened by several van der Waal forces created around the ligand backbone with respective amino acid residues of the receptor. However, ascorbic acid demonstrated a higher affinity and thus higher ligandability for LDH than do omega-3 (Figure 5). Collectively, our docking validated the inhibitory effect of omega-3/ascorbic acid against LDH.

## 4. Discussion

The liver has a vital role in the protein synthesis, metabolism of chemical toxic agents and drugs, and modulation of immunity [53]. Liver injury developed in response to alcohol, toxic agents, viral infections, and inflammatory events [54], leading to hepatic dysfunction by affecting hepatic excretion and synthesis [55], and enhanced the systemic complications such as coagulopathy, increased risk of infection, hypoglycemia, and acute kidney injury [6, 39, 56].

The mechanisms of MTX-induced hepatotoxicity are still estimated as an unclear story. It is related to the cellular and mitochondrial pathway and enhanced the proliferation of proinflammatory cytokines [57–60]. Our results revealed that the intraperitoneal injection of MTX (20 mg/kg) significantly induced the elevation of serum transaminases and ALP, hepatic LDH, and MDA while depleting the activities of antioxidants enzymes including SOD and GSH when compared to the control group. In agreement with the results obtained in this study, previous study has reported that MTX increases the inflammation [61] and generation of oxidative stress [9] due to the accumulation of lipid peroxidation leading to decrease energy formation increasing MDA levels and exerting more cellular damage [62]. ALT and AST are specific liver enzyme located in the cytosol of hepatocytes [38]. When the liver injury occurred, these enzymes are excreted on extracellular space, providing the elevation in serum blood level [38, 63]. ALP has been widely used as biomarker enzyme for assessing the integrity of endoplasmic reticulum and plasma membrane [64]. The significant elevation of these biomarker enzymes in serum of MTX-intoxicated mice could be attributed to the outflow of these enzyme from the liver into the extracellular fluid which occurs as a results of hepatic impairment. We found out that treatment of the MTX-intoxicated mice with omega-3 fatty acids or vitamin C at 100 and 200 mg/kg BW significantly attenuated the antioxidants and biochemical impairments in a dose-independent manner.

Omega-3 fatty acids play an important protective role against oxidative stress-induced DNA damage to liver tissue [26]. The decreased MDA and increased SOD and GSH levels in omega-3 fatty acid treated mice strongly reflect the free radical scavenging activities and enhance antioxidant status of MTX-intoxicated mice, a finding supported by previous clinical and experimental findings [65, 66]. Other studies also demonstrate that omega-3 has an antiapoptotic effect that protect against liver injury [67], via the inhibition of LPO, enhancing activities of mitochondrial antioxidant enzyme (GSH, SOD), reducing inflammation [27], and preserving the integrity of the hepatocytes [67]. Similarly, ascorbic acid has strong antioxidant activities by donating electrons and prevent free radical accumulation and tissue damage [68].

Coherently with the results of biochemical parameters, our histological analysis also revealed that treatment of MTX-intoxicated mice with vitamin C or omega-3 significantly attenuated the liver histological abnormalities observed in the MTX-treated mice. These histopathological findings strongly suggested the cytoprotective and hepatoprotective effects of vitamin C as well as omega-3 in amelioration of liver injury induced by MTX [69, 70]. These changes corroborated with the study of Olayaki et al. [70], which reported that omega-3 at doses of 100 and 300 mg/kg bw protects against excessive oxidative stress and exhibited a hepatoprotective effect in rats [70]. A previous study by Savran et al. [71] also demonstrated the antioxidant activities of vitamin C and its ability to reduce the hepatic oxidative tissue damage mediated by the apoptotic effect of MTX. Several other studies have also reported the antioxidants and hepatoprotective effects of vitamin C [69, 72].

The limited success rates of monotherapy and increased cases of drug resistance prompted the idea of combination therapy in treatment of liver diseases [73]. Accordingly, we observed that treatment of MTX-intoxicated mice with a combination of omega-3 fatty acids and vitamin C at lower doses exhibited higher protective effects when compared with the individual therapies. Therefore, combining omega-3 fatty acids and vitamin C appears to be a promising strategy for the treatment of drug-induced liver damage. In line with our findings, previous studies have reported the combination of the two drugs synergized to achieve enhanced hepatoprotective effect in nonalcoholic fatty liver diseases [74, 75].

Molecular docking is a widely use tool for modeling the potential binding interactions of a drug candidate with a target protein [51, 76, 77]. Our molecular docking study revealed that both ascorbic acid and omega-3 docked well to the binding cavity of LDH with the high binding affinities of –5.20 and –4.50 kcal/mol, respectively. Ascorbic acid is bounded by four hydrogen bonding to the HIS192 (2.04 Ӑ), ASN137 (2.62 Ӑ), VAL135 (2.10 Ӑ), and SER160 (2.89 Ӑ) residues of LDH binding cavity, while the hydrogen bonding with THR247 (2.12 Ӑ) of the LDH binding site exists between LDH-omega complex. Interestingly, all the conventional hydrogen interactions between LDH and ascorbic acid/omega-3 occur at very close proximity (<3.0 Ӑ). Omega-3 is also bound to LDH by several alkyl and hydrophobic contacts. In addition, LDH-ascorbic acid/omega-3 complexes were strengthened by several van der Waal forces created around the ligands backbone with respective amino acid residues of the receptor. However, ascorbic acid demonstrated a higher affinity and thus higher ligandability for LDH than do omega-3 (Figure 5). Collectively, our docking validated the inhibitory effect of omega-3/ascorbic acid against LDH.

## 5. Conclusions

The findings of this study demonstrated that the use of pretreatment with omega-3 and vitamin C provides significant hepatoprotective capacity against methotrexate-induced liver injury. This was observed by improving the oxidative damage of the liver through the depletion the serum level of MDA and enhancement of total antioxidant capacity, manifested by elevated serum level of SOD and GSH, reducing the level of intracellular liver enzyme ALT (which was the most prominent enzyme responsible for monitoring liver function). Furthermore, the current study illustrated the more synergistic effect of using a pretreatment combination of omega-3 (100 mg/kg) and vitamin C (100 mg/kg) to improve the oxidative tissue damage and liver functions affected by MTX-induced liver injury.

## Figures and Tables

**Table 1:** Experimental design and drug treatment timeline.

Groups	Designations	Treatment received (days 1-9)	Treatment received (day 10 only)
Group 1	Control	0.9% saline/day (po)	0.9% saline/day (I.P)
Group 2	MTX	0.9% saline/day (po)	20 mg/kg MTX (I.P.)
Group 3	Omega-3	100 mg/kg bw/day omega-3 (po)	20 mg/kg MTX (I.P.)
Group 4	Omega-3	200 mg/kg bw/day omega-3 (po)	20 mg/kg MTX (I.P.)
Group 5	Vitamin C	100 mg/kg bw/day vitamin C (po)	20 mg/kg MTX (I.P.)
Group 6	Vitamin C	200 mg/kg bw/day vitamin C (po)	20 mg/kg MTX (I.P.)
Group 7	Combination	Omega-3 (100 mg/kg bw/day) and vitamin C (100 mg/kg bw/day) (po)	20 mg/kg MTX (I.P.)

**Table 2:** Assessment of acute liver injury according to ranking tissue lesion severity from 0 to 3.

Score components		GR. A	GR. B	GR. C	GR. D	GR. E	GR. F	GR. G
Cellular necrosis	Score	0	+++	+	++	0	0	0
	Extent	No	>60%	<20%	>30%	No	No	No
Inflammatory cell infiltration	Score	0	+++	+	++	0	0	0
	Extent	No	>60%	<20%	>30%	No	No	No
Sinusoid dilatation	Score	0	0	+	0	+	0	+/-
	Extent	No	No	<20%	No	<20%	No	<5%
Fat droplet accumulation	Score	0	0	0	0	+	0	0
	Extent	No	No	No	No	<20%	No	No
Degenerative changes of hepatocyte cells	Score	0	+++	0	++	0	+	+/-
	Extent	No	>60%	No	>30%	No	<20%	<5%

No histopathological changes score (0), very mild histopathological changes in <5% of fields score (+/-), mild histopathological changes in <20% of fields score (+), moderate histopathological changes in 20 to 60% of fields score (++), and severe histopathological changes in >60% of fields score (+++). GR. A = control group; GR. B = MTX-injected group; GR. C and D = omega-3 pretreatment group in graded doses (100 mg and 200 mg); GR. E and F = vitamin C pretreatment group in graded doses (100 mg and 200 mg); GR. G = concomitant pretreated group with omega-3 (100 mg/kg) and vitamin C (100 mg/kg) for 10 days (each group contain 7 mice).

## Data Availability

The raw data and materials in this study can be made available upon reasonable request.
